# Supplementary material for: The effects of an evidence- and theory-informed feedback intervention on opioid prescribing for non-cancer pain in primary care: A controlled interrupted time series analysis
Source: PLoS Med. 2021 Oct 4;18(10):e1003796. doi: 10.1371/journal.pmed.1003796 (PMC8489725; doi:10.1371/journal.pmed.1003796)
Supplement: S1 Text — (PDF) [file pmed.1003796.s001.pdf]

Appendix 1: TIDieR checklist for the CROP Intervention, incorporating the reporting and design elements of audit and feedback interventions recommendations

1. **Brief name: Provide the name or a phrase that describes the intervention**

The Campaign to Reduce Opioid Prescribing (CROP)

2. **Why: Describe any rationale, theory, or goal of the elements essential to the intervention**

Audit and feedback (A&F) aims to improve patient care by reviewing health care performance against explicit standards. Ideally, where a discrepancy is detected, changes are implemented at an individual, team, and/or service level. Our earlier work identified the scale of the opioid prescribing problem for chronic pain in primary care. Given accumulating evidence of harm, reversing the current trend in opioid prescribing would benefit a substantial at-risk population. Such a reversal requires both individual and population-based approaches. Guidelines recommend both pharmacological and behavioural approaches to pain management in individuals. However, there is little evidence on the effectiveness of interventions to reduce opioid prescribing in individual patients with chronic pain. We therefore developed a feedback intervention targeting prescribing of opioids for chronic pain in general practice.

3. **What (materials): Describe any physical or informational materials used in the intervention, including those provided to participants or used in intervention delivery or in training of intervention providers. Provide information on where the materials can be accessed (for example, online appendix, URL)**

The intervention entailed general practices receiving a comparative and practice-individualised feedback report on the number of their patients taking opioid medication and for patient groups at risk of long-term or strong opioid prescribing, and prescribing in relation to all other practices receiving the reports. From report two onwards, comparison to own prescribing at the start of the intervention was included. Aggregated patient data for each practice were extracted from electronic health record systems and reports were sent within two weeks of extraction. Individual prescriber-level data were not available and no patient outcome data were included. Data excluded those with coded cancer, palliative care or drug dependence. The intervention also highlighted patient groups at higher risk of long-term or stronger opioid prescribing such as those with mental health diagnoses, concurrent benzodiazepine use and the elderly who are at higher risk of adverse effects. We could not use Read codes to reliably define clinical diagnostic categories (e.g. chronic pain) given highly variable coding practice which would have missed large numbers of patients if we tried to specify codes. The reports presented the data as total patient numbers and percentage of patient population, and in a bar chart that highlighted practices in the same area (CCG) and the lowest quartile of prescribers. Reports incorporated evidence-informed behaviour change techniques, such as specific recommendations for action and an action plan to complete, and the 15 recommendations for audit and feedback, designed to enhance effectiveness.

4. **What (procedures): Describe each of the procedures, activities, and/or processes used in the intervention, including any enabling or support activities**

Five copies of the feedback reports were sent to each practice bimonthly.

5. **Who provided: For each category of intervention provider (for example, psychologist, nursing assistant), describe their expertise, background and any specific training given**

Reports were sent by the West Yorkshire Research and Development team on behalf of the ten West Yorkshire CCGs and the research team at the University of Leeds. The reports were written by a GP and clinical lecturer in primary care and reviewed by the research team.

6. **How: Describe the modes of delivery (such as face to face or by some other mechanism, such as internet or telephone) of the intervention and whether it was provided individually or in a group**

Five copies of the feedback reports were sent by post to each practice, addressed to the practice manager, from April 2016 to March 2017 by the research team. Eight of the ten medicines optimisation leads for the CCGs also sent the reports by email to the practice managers, the other two CCG medicine optimisation leads loaded them onto the CCG intranet for practices to access.

7. **Where: Describe the type(s) of location(s) where the intervention occurred, including any necessary infrastructure or relevant features**

316 out of 317 general practices in the ten CCGs of West Yorkshire received the feedback reports. One practice did not receive the reports as they did not have a data sharing agreement with their CCG.

8. **When and how much: Describe the number of times the intervention was delivered and over what period of time including the number of sessions, their schedule, and their duration, intensity or dose**

Feedback reports delivered bimonthly from April 2016 to March 2017 with a total of six reports sent.

9. **Tailoring: If the intervention was planned to be personalised, titrated or adapted, then describe what, why, when, and how**

N/A

10. **Modifications: If the intervention was modified during the course of the study, describe the changes (what, why, when, and how)**

The electronic health record searches used to collect data were made available through the CCG Medicine Optimisation Teams and could be accessed by practices on SystmOne and Emis Web with instructions on how to find the searches included in the final three reports.

11. **How well (planned): If intervention adherence or fidelity was assessed, describe how and by whom, and if any strategies were used to maintain or improve fidelity, describe them**

Intervention adherence and fidelity was not assessed.

12. **How well (actual): If intervention adherence or fidelity was assessed, describe the extent to which the intervention was delivered as planned**

Intervention adherence and fidelity was not assessed.
