# Supplementary material for: The effects of an evidence- and theory-informed feedback intervention on opioid prescribing for non-cancer pain in primary care: A controlled interrupted time series analysis
Source: PLoS Med. 2021 Oct 4;18(10):e1003796. doi: 10.1371/journal.pmed.1003796 (PMC8489725; doi:10.1371/journal.pmed.1003796)
Supplement: S2 Text — The baselayer of the map used in this file is from https://commons.wikimedia.org/wiki/File:England_Clinical_Commissioning_Group_(CCG)_Map_(Labelled).svg. (PDF) [file pmed.1003796.s002.pdf]

Appendix 2: Sample practice report

[redacted]

[redacted]

Can your practice review and reduce opioid prescribing?

Dear Practice Manager and colleagues,

Many doctors and professional bodies are concerned about rising opioid prescribing in general practice. Much of this prescribing is for chronic non-cancer pain, which is often difficult to treat. However, there is little evidence for the effectiveness of opioids in chronic pain but accumulating research indicating that the harms of opioids to patients can outweigh benefits As well as addiction, prescribed opioids are associated with higher risks of hospitalisation and premature death.

Therefore, we are undertaking a major Campaign for the Reduction of Opioid Prescribing (CROP) across West Yorkshire to reduce opioid prescribing for chronic pain. We recommend that all general practices review and, where clinically appropriate, reduce opioid prescribing. You will receive regular feedback to your practice on your current levels of opioid prescribing. This is the first report for your practice.

Doctors' prescriptions are killing people, and this is an international problem, with rapid increases in opioid prescriptions in Canada, Australia, Germany, and the UK.

We could blame the marketing of big pharma, but the truth is that these deaths are the responsibility of doctors. We must put it right.

Des Spence, GP. The painful truth: deaths and misuse of prescribed drugs BMJ 2011; 343 :d7403

We invite you to review your practice's prescribing of opioids and ways of avoiding initiation of long term opioid prescribing.

Please distribute this report to all prescribers within your practice team and identify a time to discuss it at a practice meeting.

The CCG will provide ten copies of this report for your team, if you require more please contact:

Yours sincerely,

Paul Carder,
Head of West Yorkshire Research and Development

[redacted]

What are the troubling trends in opioid prescribing?

- 110 general practices from Bradford and Leeds took part in a recent study.(1) This showed a marked increase in all opioid prescribing – even after excluding patients with known cancer or drug dependence.
- The proportion of all adult patients prescribed a weaker opioid at least once almost **doubled** over seven years whilst the proportion prescribed a stronger opioid has increased over **six-fold**. (1)
- There was a **10-fold variation** in opioid prescribing between practices which could not be explained away by many patient or practice factors (e.g. deprivation).(1)
- Long-term prescribing may follow hospitalisation or a secondary care consultation but usually starts in primary care.(2)

Why review opioid prescribing?

Whilst opioids provide useful and effective analgesia in the short term for acute pain following trauma (including surgery) and cancer pain, the safety and efficacy of opioids for chronic non-cancer pain is uncertain. They can cause problems of tolerance, dependence and addiction.(3)

There is concern that patients with chronic pain are being moved up the World Health Organisation 'analgesic ladder,' originally developed for cancer pain, towards potent opioids inappropriately and without considering alternatives to medication.(4) The benefits of opioid treatment for your patients must be balanced against burdens of long term use as prescribing for chronic pain often continues for months or years.(5)

Prescribing

Comprehensive assessment is important; patients with depression, anxiety, or other psychiatric or psychological co-morbidity will need additional support and monitoring to avoid problem drug use.

There is considerable scope to reduce new prescribing, and prescribing in selected patients. **Think twice** before prescribing an opioid.

Goals of therapy should be agreed before a trial of opioids; complete pain relief is unlikely, and treatment success is demonstrated by the patient becoming able to do things that the pain currently prevents. Agree a **trial period** with the patient and **review treatment regularly**, especially if there are any concerns.

Efficacy and **adverse effects are similar** for all opioids, though patients may tolerate one drug better than another.

Requests for **dose increase** need careful evaluation.

For the problem of chronic pain; more difficult and complex patients still require more time intensive or **specialist input**.

Potential harms

80% of patients taking opioids will experience at least one **adverse effect** e.g. constipation, nausea, itching, dizziness, hospitalisation and death.

Prescribed opioids are associated with increased **psychosocial problems, hospitalisation and mortality.**

Opioid toxicity (sedation, slow respiration) is more likely with increasing age, co-morbidity, co-prescribing, and if taken with alcohol or illicit drugs.

Opioids have **long term** endocrine and immunological effects e.g. reduced libido, depression, susceptibility to infections.

Withdrawal symptoms occur if opioid is stopped/ dose reduced abruptly e.g. sweating, yawning, abdominal cramps. This is common with Tramadol even after a short course.

Addiction is characterised by impaired control and over use, craving and continued use despite harm.

Opioid induced hyperalgesia may occur: pain becomes more diffuse and qualitatively different from pre-existing pain.

How is your practice doing?

Medical Centre

Achievement in participating practices across West Yorkshire

The graph below demonstrates:

Your practice (black bar) and number of patients of the practice population prescribed opioids (358) in the last 8 weeks; a lower value indicates better clinical practice. The audit data exclude patients with a cancer diagnosis, on the palliative care register or drug addiction diagnosis.

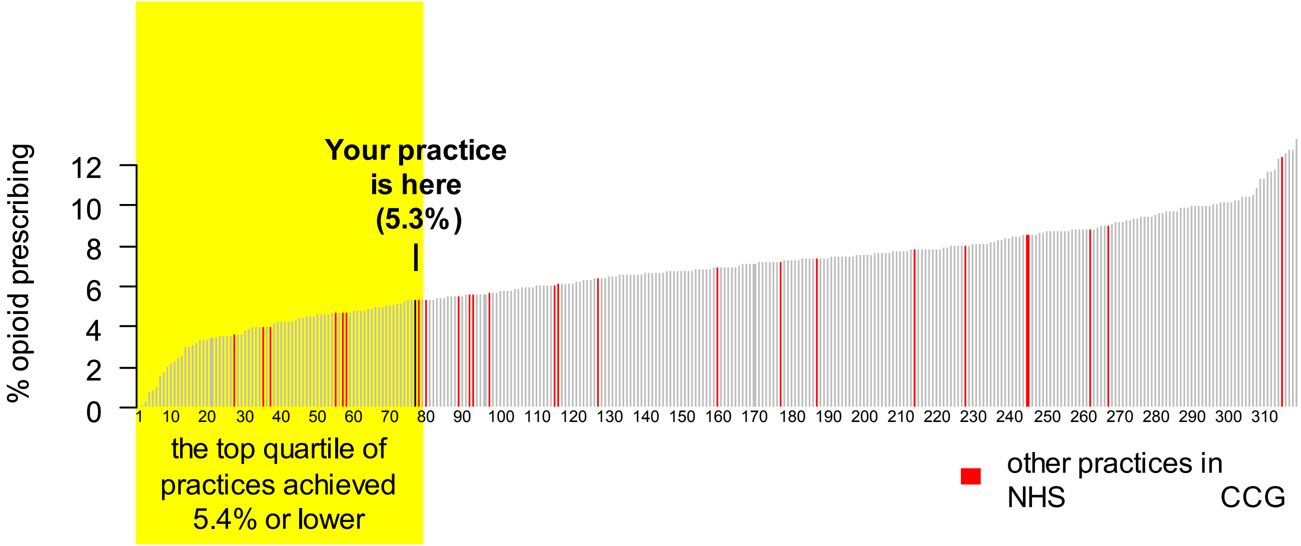

- Achievement throughout West Yorkshire overall (range 0 to 13.4%)
- The best achieving practices within West Yorkshire (yellow box – achieving 5.4% or below)
- Other practices within NHS CCG (red bars)

Your practice achievement on individual indicators:

Risk Factor	Number of patients
Prescribed strong opioids	12
Men aged under 50 years and prescribed strong opioids	0
Patients aged over 75 years and prescribed strong or weak opioid	66
Women aged over 65 years and prescribed strong or weak opioid	71
Polypharmacy (on 10 or more repeat prescriptions) and prescribed strong or weak opioid	81
All mental health diagnoses and prescribed strong or weak opioid	170
Taking antidepressant and a strong or weak opioid	57
Taking benzodiazepines and a strong or weak opioid	4

What next?

Can you halt the increase in opioid prescribing at your practice?

Make a plan about what your individual practice team members want to do, when and with whom. It may involve one or more of the following:

Think twice about prescribing opioids for the first time. Consider alternative forms of analgesia and patient support if the condition being prescribed for is likely to be long term or in a high risk patient.

At **medication reviews** check over-adherence, indication and assess for behaviours that may indicate problem usage.

Identify a practice **opioid champion** who will lead on this work and review relevant prescribing guidelines.

Consider **allocating records** for review within the team to the patient's usual GP or to a pharmacist for review and follow-up (if necessary) by usual GP. Could administrative staff identify and code patients?

Review your **progress** in light of further feedback we will send you later.

Medical Centre team plan of action is to :

- What are we going to do (e.g. which risk factors would you like to review if any)?

- When are we going to do it (opportunistic, systematic, a combination or another time)?

- Who will be involved (GPs, pharmacist, administrative staff)?

Frequently Asked Questions

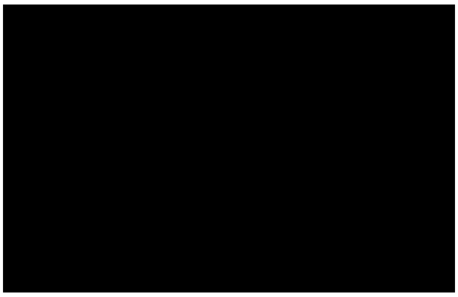

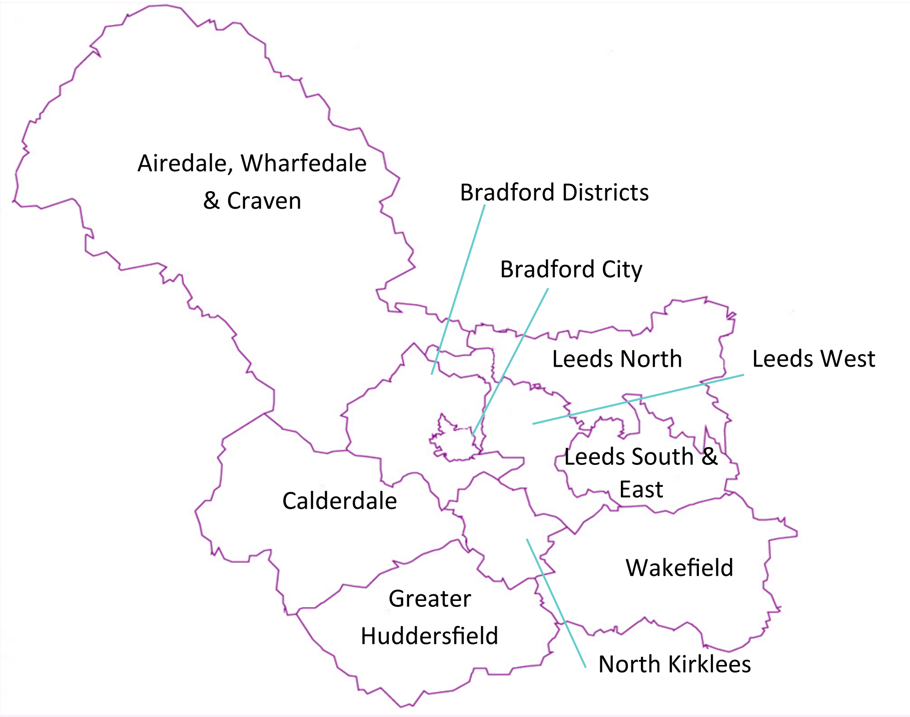

What is the Campaign for Reduction in Opioid Prescribing (CROP)?

CROP is a collaboration between all medicines management leads of the 10 CCGs of West Yorkshire and a multidisciplinary group at the University of Leeds led by Dr Sarah Alderson, Clinical lecturer in Primary Care and sessional GP in Halifax. We aim to halt the increase in opioid prescribing in primary care through performance feedback.

We are very busy. Why should we prioritise opioid prescribing?

We know that practices are currently under a great deal of pressure and there are increasing demands within consultations. However, reducing opioid prescribing can prevent adverse events (e.g. constipation, sedation, overdose and addiction) all of which increase demands on your practice. Furthermore, learning new consultation 'scripts' (example phrases to help discuss difficult issues) to manage patient's expectations for a medicine as a "cure" for pain could reduce unnecessary consultations.

Our practice population is different and our prescribing reflects this.

Studies have shown that patient and practice factors such as deprivation, patient demographics, number and experience of GPs and QOF attainment only partially explain the 10-fold difference in prescribing between practices. Practice team and individual clinician prescribing behaviours account for the variation in levels of opioid prescribing.

What is a weak or strong opioid?

Weaker opioids include codeine (with or without paracetamol or ibuprofen), dihydrocodeine (with or without paracetamol), tramadol, pethidine, meptazinol and tapentadol. Stronger opioids comprise of diamorphine, morphine, oxycodone, fentanyl, hydromorphone, buprenorphine, pentazocine, dipipanone

How can I influence the trend for increasing opioid prescribing?

The rate of increase in the number of patients prescribed opioids could be stopped if just **1 in 20** new opioid prescriptions were not prescribed. This would potentially save the prescribing budget approximately £500,000 across West Yorkshire in the next year.

How often will I receive this feedback?

Practices will be re-audited every two months and an updated version of the feedback will be sent so you can see your practices progress in reducing opioid prescribing.

Report 1<br>June 2016	Report 2<br>August 2016	Report 3<br>October 2016	Report 4<br>December 2016	Report 5<br>February 2017	Report 6<br>April 2017

Where do these data come from?

These West Yorkshire data were extracted from SystmOne or EMIS by the CCGs on 22nd June 2016.

Why can't I produce the same numbers as the report?

It is important to remember that you may have changed patient care since we collected these data. SystmOne updates on a daily basis so it will not be possible to replicate the figures in your practice feedback reports.

References:

1. Foy, R., Leaman, B., McCrorie, C., Petty, D., House, A., Bennett, M.I., Carder, P., Faulkner, S., Glidewell, L. and West, R. 2016. Prescribed opioids in primary care: cross sectional and longitudinal analyses of influence of patient and practice characteristics. *BMJ Open.* Accepted March 2016.

2. Clarke, H., Soneji, N., Ko, D.T., Yun, L. and Wijeysundera, D.N. 2014. Rates and risk factors for prolonged opioid use after major surgery: population based cohort study.

3. Noble, M., Treadwell, J.R., Tregear, S.J., Coates, V.H., Wiffen, P.J., Akafomo, C. and Schoelles, K.M. 2010. Long-term opioid management for chronic noncancer pain. *Cochrane Database Syst Rev.* 1(1).

4. Heit, H.A. and Gourlay, D.L. 2010. Tackling the difficult problem of prescription opioid misuse. *Annals of internal medicine.* 152(11), pp.747-748.

5. Opioids for persistent pain: summary of guidance on good practice from the British Pain Society. 2012. *British Journal of Pain.* 6(1), pp.9-10.

© University of Leeds 2016
