# Supplementary material for: The effects of an evidence- and theory-informed feedback intervention on opioid prescribing for non-cancer pain in primary care: A controlled interrupted time series analysis
Source: PLoS Med. 2021 Oct 4;18(10):e1003796. doi: 10.1371/journal.pmed.1003796 (PMC8489725; doi:10.1371/journal.pmed.1003796)
Supplement: S3 Text — (PDF) [file pmed.1003796.s003.pdf]

Appendix 3: Primary outcome search terms for TPP SystmOne electronic health record system

Mandatory In
Optional In
Not In

CROP02 FINAL Aged >18, all opioids with patient & drug exclusions
CROP / Nov 17

- IN → **zzjoins *All opioids exc patients & opioid dependance drugs**
  CROP / Nov 17
  - IN → **zzjoins *All opioids exc opioid dependance drugs**
    CROP / Nov 17
    - NOT IN → **zjoins Excluded opioids L8W**
      CROP / Nov 17
      - Has medication in the 'Opioid dependence' Action Group
      - Include all drug types
      - Date of medication between 05 Oct 2017 and 30 Nov 2017
      - Including active, deceased & deducted patients
      - IN → **zjoins Patients as at Month**
        CROP / Nov 17
        - Date of death after 01 Nov 2017
        - Including active, deceased & deducted patients
        - Registered before 01 Nov 2017
        - Deducted after 01 Nov 2017
    - AND IN → **zjoins All opioids L8W**
      CROP / Nov 17
      - Has medication in the 'Opioid analgesics' Action Group
      - Include all drug types
      - Event was entered between 05 Oct 2017 and 30 Nov 2017
      - Including active, deceased & deducted patients
      - IN → **zjoins Patients as at Month**
        CROP / Nov 17
        - Date of death after 01 Nov 2017
        - Including active, deceased & deducted patients
        - Registered before 01 Nov 2017
        - Deducted after 01 Nov 2017
  - NOT IN → **zzjoins *All excluded patients**
    CROP / Nov 17
    - IN → **zjoins Cancer**
      CROP / Nov 17
      - Has a Read code in the CAN (Codes for relevant malignancies) QOF cluster
        Show read codes in cluster CAN.
      - Selecting only the earliest matching code
      - Date of Read code before 01 Nov 2017
      - Including active, deceased & deducted patients
      - IN → **zjoins Patients as at Month**
        CROP / Nov 17
        - Date of death after 01 Nov 2017
        - Including active, deceased & deducted patients
        - Registered before 01 Nov 2017
        - Deducted after 01 Nov 2017
    - OR IN → **zjoins Substance dependance**
      CROP / Nov 17
      - Has a Read code in...Codes and Children:
        Drug dependence (E24..)
        Combined opioid with other drug dependence (E248.)
        Drug dependence NOS (E24z.)
        Controlled drug dependence (X00S3)
        Active drug dependence (X00S4)
        Opioid type drug dependence (XE1YR)
        Other specified drug dependence (XE1YW)
      - Selecting only the earliest matching code

- Date of Read code before 01 Nov 2017
- Including active, deceased & deducted patients

IN → **zjoins Patients as at Month**
CROP / Nov 17

- Date of death after 01 Nov 2017
- Including active, deceased & deducted patients
- Registered before 01 Nov 2017
- Deducted after 01 Nov 2017

OR IN → **zjoins Palliative care**
CROP / Nov 17

- Has a Read code in the PC1 (Palliative care codes (PALCARE)) QOF cluster
  Show read codes in cluster PC1.
- Selecting only the earliest matching code
- Date of Read code before 01 Nov 2017
- Including active, deceased & deducted patients

IN → **zjoins Patients as at Month**
CROP / Nov 17

- Date of death after 01 Nov 2017
- Including active, deceased & deducted patients
- Registered before 01 Nov 2017
- Deducted after 01 Nov 2017

AND IN → **zCROP11 Aged > 18**
CROP / Nov 17

- Born between 01 Dec 1906 and 30 Nov 1999
- Matching to GMS patients
- Including active, deceased & deducted patients

IN → **zjoins Patients as at Month**
CROP / Nov 17

- Date of death after 01 Nov 2017
- Including active, deceased & deducted patients
- Registered before 01 Nov 2017
- Deducted after 01 Nov 2017
