# Supplementary material for: The effects of an evidence- and theory-informed feedback intervention on opioid prescribing for non-cancer pain in primary care: A controlled interrupted time series analysis
Source: PLoS Med. 2021 Oct 4;18(10):e1003796. doi: 10.1371/journal.pmed.1003796 (PMC8489725; doi:10.1371/journal.pmed.1003796)
Supplement: S4 Text — (PDF) [file pmed.1003796.s004.pdf]

Appendix 4: Linear Multi-level Mixed effect model

Assumptions for all outcomes:

i) Linear change in outcome over time – linear model
ii) Each change in outcome over time differs for each practice – linear mixed effect model
iii) The practices are within CCG areas that might have area-level similarities – linear multi-level mixed effect model.

We fitted a three-level model with the following random effects: a random intercept and random slope on month at the practice level, and a random intercept at the CCG level, with practice nested within CCG.

We wished to estimate the change (and differences) in the outcomes over time between the intervention and control practices, for each of the 3 intervention periods within a single model. Therefore, an interaction term as a fixed effect was included – intervention (control/intervention), the three intervention time periods (pre-intervention/intervention/post-intervention) and month (July 2013 to December 2017).

Practice level characteristics were included as fixed effects as did not change with time in the dataset.

Then the number of adults prescribed opioid per 1,000 adults represented as y_{ijk} at month *i*, *j* practice and *k* CCG modelled as:

$$
\begin{aligned}
y_{ijk} = \beta_0 &+ \beta_1(intervention_{ijk}) + \beta_2\left(month_{ijk}\right) + \ \beta_2\left(intervention_{ijk} \times month_{ijk}\right) \\
&+ \beta_3\left(intervention\ period_{ijk}\right) + \beta_4\left(intervention_{ijk} \times intervention\ period_{ijk}\right) \\
&+ \beta_5\left(intervention\ period_{ijk} \times month_{ijk}\right) \\
&+ \beta_6\left(intervention_{ijk} \times intervention\ period_{ijk} \times month_{ijk}\right) + \beta_7(female\_percent_{ijk}) \\
&+ \beta_8(pat\_exper_{ijk}) + \beta_9(LTC\_pc_{ijk}) + \beta_{10}(QOF_{ijk}) + \beta_{11}(IMD_{ijk}) + u_{0j} + u_{1j}\left(month_{ijk}\right) \\
&+ v_{ojk} + e_{ijk}
\end{aligned}
$$

e_{ijk}, u_{0j} and v_{ojk} have mean 0, are normally distributed and are uncorrelated across the three levels.
