# Supplementary material for: The effects of an evidence- and theory-informed feedback intervention on opioid prescribing for non-cancer pain in primary care: A controlled interrupted time series analysis
Source: PLoS Med. 2021 Oct 4;18(10):e1003796. doi: 10.1371/journal.pmed.1003796 (PMC8489725; doi:10.1371/journal.pmed.1003796)
Supplement: S5 Text — (PDF) [file pmed.1003796.s005.pdf]

Appendix 5: Sensitivity Analysis

All sensitivity analysis was conducted on the main outcome – number of adults prescribed opioid per 1,000 adults (electronic health record data and denominator) adjusted LMM.

1. Assess impact of outliers on model estimates

To assess the impact of outliers, predicted values with residuals more than 2 or -2 were removed and the same adjusted LMM was re-run for the main outcome. The estimates (Table S1) were similar to that estimated in Table 3 in the main paper, and so outliers do not appear to have an impact on the LMM estimates for the main outcome.

2. Comparing models

Rather than a LMM where the outcomes were modelled as the rate, another approach could be to model the count of the number of adults prescribed opioid and the practice size as the exposure using multilevel mixed-effects Poisson and Negative Binomial regression models. Comparisons of Akaike information criterion (AIC) and Bayesian information criterion (BIC) values for Multilevel mixed-effects Poisson and Negative Binomial regression models and the adjusted LMM (all without CCG level due to convergence issues) for the main outcome indicated that the LMM was the most appropriate fit to the data.

TABLE S1: Model Fit Statistics

Mixed effects Model*	AIC	BIC
Linear – rate as outcome	161700.4	161857.2
Poisson – count as outcome	580299.1	580440.3
Negative Binomial – count as outcome	225461.5	225610.6

* adjusted by % Female; QOF, Quality Outcomes Framework; Patient Experience; % LTC, Long Term Conditions; and IMD, Index of Multiple Deprivation
