# Supplementary material for: The effects of an evidence- and theory-informed feedback intervention on opioid prescribing for non-cancer pain in primary care: A controlled interrupted time series analysis
Source: PLoS Med. 2021 Oct 4;18(10):e1003796. doi: 10.1371/journal.pmed.1003796 (PMC8489725; doi:10.1371/journal.pmed.1003796)
Supplement: S6 Text — (PDF) [file pmed.1003796.s006.pdf]

TABLE S2: Mean Number of Adults prescribed Opioid per 1,000 adults: multi-level linear model: electronic health record data and denominator – residuals (±2) removed

Timeframe	Month	Mean no. of adults prescribed opioid per 1,000 adults			Mean change per month, over the timeframe		
		Control (n=130)	Intervention (n=213)	Difference	Control (n=130)	Intervention (n=213)	Difference
**Mean number of adults prescribed opioid per 1,000 adults – adjusted[a]**							
**Pre-intervention**	2013m9	54.3 (45.9 ,62.6)	57.6 (49.7 ,65.5)	3.3 (-8.2 ,14.8)	0.4 (0.34 ,0.47)	0.18 (0.13 ,0.23)	-0.23 (-0.31 ,-0.14)
	2016m3	66.3 (58.3 ,74.4)	62.8 (55.1 ,70.5)	-3.5 (-14.6 ,7.6)			
**Intervention**	2016m4	61.4 (53.4 ,69.4)	63.2 (55.5 ,70.9)	1.8 (-9.4 ,12.9)	0.49 (0.39 ,0.59)	0 (-0.08 ,0.07)	-0.49 (-0.62 ,-0.37)
	2017m3	66.8 (58.8 ,74.8)	63.1 (55.5 ,70.8)	-3.6 (-14.8 ,7.5)			
**Post-intervention**	2017m4	64 (56 ,72)	61.2 (53.5 ,68.8)	-2.8 (-14 ,8.3)	0.18 (0.08 ,0.28)	0.07 (0 ,0.15)	-0.1 (-0.23 ,0.02)
	2018m3	65.9 (57.8 ,74)	62 (54.2 ,69.7)	-4 (-15.2 ,7.3)			

[a] adjusted by % Female; QOF, Quality Outcomes Framework; Patient Experience; % LTC, Long Term Conditions; and IMD, Index of Multiple Deprivation
