# Supplementary material for: The effects of an evidence- and theory-informed feedback intervention on opioid prescribing for non-cancer pain in primary care: A controlled interrupted time series analysis
Source: PLoS Med. 2021 Oct 4;18(10):e1003796. doi: 10.1371/journal.pmed.1003796 (PMC8489725; doi:10.1371/journal.pmed.1003796)
Supplement: S7 Text — (PDF) [file pmed.1003796.s007.pdf]

[redacted]

Final

Statistical Analysis Plan

Campaign to Reduce Opioid Prescribing

Controlled Interrupted Time Series Analysis

Version 1.0

November 2019

Amendments

<< This section details amendments to the analysis plan after sign off. A description and reasons for the amendment must be provided alongside the version change numbers >>

1. Introduction

The use of opioid medicines in the treatment of chronic, non-cancer pain is questionable given limited evidence of efficacy and growing evidence of harm. Concerning trends and variations in opioid prescribing for non-cancer pain in general practice were found in earlier work. Following dissemination, the ten clinical commissioning groups (CCGs) in West Yorkshire identified opioid prescribing as a priority for action. Subsequently, we advised on the design and delivery of a feedback intervention to reduce opioid prescribing in general practice – the 'Campaign to Reduce Opioid Prescribing (CROP)'.

316 practices across the ten CCGs received bimonthly feedback reports on their opioid prescribing over a 12-month period (April 2016 to March 2017). These reports included a range of embedded behaviour change techniques intended to enhance effectiveness. We wish to evaluate the effectiveness of this 'natural experiment' using an interrupted time series analysis (iTSA) approach.

1.1 Background

1.1.1 THE PROBLEM

There is international concern over rising trends in opioid prescribing,[1-5] largely attributed to prescribing for chronic non-cancer pain.[6] It is likely that patients with chronic pain are inappropriately being moved up the World Health Organisation 'analgesic ladder,' originally developed for cancer pain, without considering alternatives to medication.[7-9]

Opioids have short-term efficacy in neuropathic and musculoskeletal pain, however efficacy decreases with long-term use and dependence can make treatment withdrawal challenging.[10] Prescribed opioids, even weaker opioids such as codeine, are associated with psychosocial problems, hospitalisation and increased mortality.[11-14]

Long-term prescribing usually starts in primary care.[15] Our earlier work identified marked increases in opioid prescribing across 111 general practices in Leeds and Bradford, even after excluding patients with coded cancer or drug dependence.[16] Our findings were consistent with those elsewhere in the UK.[5] The proportion of all patients prescribed a weaker opioid almost doubled over seven years, whilst stronger opioid prescribing increased over six-fold. Around one in 20 adults are prescribed long-term opioids; nearly one in 100 are prescribed stronger opioids. We identified substantial, poorly explained variation amongst practices in long-term and stronger opioid prescribing, particularly in the likelihood of stepping up to stronger opioids.

1.1.2 THE NEED FOR ACTION

We have shown patient and professional dissatisfaction with current chronic pain management and prescribing of long-term opioids.[17] Patients have unmet needs for pain relief, an explanation of their symptoms and help to improve or maintain their quality of life. GPs expressed dissatisfaction with their approach to chronic pain management, be it with their own consultation skills, the drugs they prescribe, or the constraints of everyday practice.[17]

Given accumulating evidence of harm, reversing the current trend in opioid prescribing would benefit a substantial at-risk population. Such a reversal requires both individual and population-based approaches. Guidelines recommend both pharmacological and behavioural approaches to pain management in individuals.[18-21] However, there is little evidence on the effectiveness of interventions to reduce opioid prescribing in individual patients with chronic pain.[22]

There is therefore a pressing need for a population-based approach to reduce opioid prescribing in general practice. The large variations we identified suggests that much opioid prescribing is driven by GP habits and norms rather than patient need and evidence of benefit. An 'upstream' approach can reduce prescribing by (i) prompting GPs to think twice before initiating prescribing opioids, especially in patient groups at higher risk of long-term or stronger opioid prescribing, and (ii) encouraging medication reviews of patients currently prescribed opioids who may not be benefiting from them.

1.1.3 THE POTENTIAL OF AUDIT AND FEEDBACK

Audit and feedback (A&F) aims to improve patient care by reviewing health care performance against explicit standards. Ideally, where a discrepancy is detected, changes are implemented at an individual, team, and/or service level. A Cochrane Review indicated that A&F can improve healthcare processes by a median of 4.3%[31]. Effects vary markedly between studies (interquartile range 0.5% to 16%). Meta-regression indicated that feedback is more effective when provided more than once and when it includes both explicit targets and an action plan. Even relatively modest effects can still translate into substantial population impacts. Furthermore, the availability of routinely collected data means that feedback can be delivered efficiently at scale. We know of no UK studies which have evaluated A&F to reduce opioid prescribing in primary care.

1.1.4 CAMPAIGN TO REDUCE OPIOID PRESCRIBING

Our earlier work alerted our ten West Yorkshire CCGs to the scale of the opioid prescribing problem. We therefore developed a feedback intervention targeting prescribing in general practice. We drew upon our track record of analysing routinely collected data, well-received earlier NIHR programme work in developing enhanced feedback interventions for general practice[32] and our wider expertise in A&F.[33-37] The intervention entails all practices in West Yorkshire receiving a comparative and practice-individualised bimonthly feedback report on their overall opioid prescribing and for patient groups at risk of long-term or strong opioid prescribing, and prescribing in relation to all other 316 West Yorkshire practices. This population level intervention targets all patients aged over 18 who are taking opioid medication, particularly for long-term pain of any cause. It excludes those with coded cancer or drug dependence. The intervention also highlights patient groups at higher risk of long-term or stronger opioid prescribing such as those with mental health diagnoses, concurrent benzodiazepine use and the elderly who are at higher risk of adverse effects. We could not use Read codes to reliably define clinical diagnostic categories (e.g. chronic pain) given highly variable coding practice.[1] We would miss large numbers of patients if we tried to specify codes.

This intervention differs from usual care in several respects. It makes a more sophisticated use of data than audits run by CCGs or individual practices. Comparative prescribing data usually used by CCGs are relatively crudely based on dispensed prescription ('PACT' data) with attempted weighting for population characteristics (e.g. 'STAR PUs'). Our intervention incorporates more in-depth analyses related to risk factors for longer or stronger opioid prescribing and access to our searches allows practices to identify and review individual patients. It also incorporates evidence-informed behaviour change techniques, such as specific recommendations for action, designed to enhance effectiveness.[38] The 316 practices across the ten CCGs received bimonthly feedback reports on their opioid prescribing over a 12-month period (April 2016 to March 2017).

1.2 DESIGN

CROP iTSA is a controlled interrupted time series analysis comparing opioid prescribing in practices who received the enhanced feedback intervention to CCGs in North, East and South Yorkshire (controls). Given that randomisation was not feasible for an existing area-wide service development, we propose a controlled interrupted time series analysis to evaluate population-level effects. This quasi-experimental design exploits existing routine clinical data, and has been used previously to examine the impact of policy and quality improvement initiatives in primary care.[1, 3-5] It is an acceptably robust design where randomisation is not feasible, producing results largely concordant with those of cluster randomised trials.[6-8]

We recognise that effects may vary according to practice and patient characteristics, and how practices perceive and act upon feedback.

1.3 Aims

To evaluate an enhanced feedback intervention to reduce opioid prescribing in general practice.

1.3.1 Primary Objective

To evaluate the effectiveness of the enhanced feedback intervention in reducing the number of patients taking opioid medication (excluding those with coded cancer, palliative care or drug dependency) in primary care.

1.3.2 Key Secondary Objectives

To evaluate any wider effects on non-opioid prescribing, explore associations between any intervention effects and practice or patient characteristics, and the effectiveness in reducing the overall number of opioid medications prescribed.

1.3.3. Secondary Objectives

1. To evaluate the effectiveness of the enhanced feedback intervention in:
    a. Reducing the number of patients taking 'strong' opioid medications
    b. Reducing the number of patients taking opioid medication who are at higher risk of adverse effects or long-term or stronger opioid prescribing including:
        i. Men aged between 18 and 50 taking strong opioids
        ii. Women aged 65 and over
        iii. Patients aged 75 and over
        iv. Patients with polypharmacy (10 or more medications on repeat prescription)
        v. Patients taking concurrent antidepressants
        vi. Patients taking concurrent benzodiazepines
        vii. Patients with mental health diagnoses

2. To evaluate any wider effects of the enhanced feedback intervention on other non-opioid analgesic prescribing and referrals including:
    a. The number of patients prescribed Non-Steroidal Anti-Inflammatories (NSAIDs)
    b. The number of patients prescribed gabapentinoids (gabapentin and pregabalin)
    c. The number of patients referred to musculoskeletal (MSK) clinics

3. To explore associations between any intervention effects and practice or patient characteristics, including:
    a. Practice list size
    b. Proportion of females in practice
    c. Practice population who are nursing home residents
    d. Practice patient experience score
    e. Index of Multiple Deprivation (IMD) score
    f. Proportion of practice population in employment
    g. Practice QOF score
    h. Practice CQC score

4. To evaluate the effectiveness of the enhanced feedback intervention in reducing the number of opioid medications prescribed.

1.4 Sample Size

The intervention group comprises 316 practices in the ten CCGs of West Yorkshire:

- NHS Airedale, Wharfdale and Craven CCG
- NHS Bradford City CCG
- NHS Bradford Districts CCG
- NHS Calderdale CCG

- NHS Greater Huddersfield CCG
- NHS Leeds North CCG
- NHS Leeds South and East CCG
- NHS Leeds West CCG
- NHS North Kirklees CCG
- NHS Wakefield CCG

The control group comprises XXX practices in South Yorkshire and North Yorkshire, Humber and North Lincolnshire:

- NHS East Riding of Yorkshire CCG
- NHS Hull CCG
- NHS North East Lincolnshire CCG
- NHS North Lincolnshire CCG
- NHS Sheffield CCG

2. Data

2.1 Data Monitoring

Data will be monitored for quality and completeness by the West Yorkshire Research and Development (R&D) team. Missing data will be chased until it is received, confirmed as not available, or the study is at final analysis. Every effort will be made to ensure as much data as possible are available, and that reasons for unobtainable data are obtained.

2.2 Data Sources

2.2.1 Objectives 1 and 2:

Routinely collected, practice aggregated patient data will be extracted centrally from electronic health records (EHRs) via West Yorkshire R&D team. Data will be stripped of practice identifiers and obtained in monthly epochs, spanning a 5-year period and including: the 3 years prior to the feedback intervention (April 2012 – March 2016); the year of the intervention (April 2016 – March 2017); and the 12 months after the intervention has ceased (April 2017 – March 2018).

2.2.2 Objective 3:

Practice demographics data will be extracted from the Public Health website, Fingertips (https://fingertips.phe.org.uk/profile/general-practice) for the year 2018.

2.2.3 Objective 4:

Routinely collected prescription data, aggregated by practice, will be extracted from OpenPrescribing.com for the same time periods as Objectives 1 and 2.

3. Data Analysis

3.1 Primary Analysis

The potential change in the number of patients taking opioid medication will be assessed using an ITS model. This type of model is useful for examining whether a change occurred in a data series following the introduction of an intervention. It is particularly useful where it is not possible to randomise but where it is of interest to assess the impact of an intervention following its introduction across a population. It allows for the impact to be measured, having adjusted for seasonality in the data series. Whilst seasonality is unlikely to be an issue in opioid prescribing, the presence of seasonal fluctuations will be investigated and if necessary adjusted for using a sine function.

By fitting separate coefficients it is possible to model whether there was sudden change (break in the data series) or a gradual change (change in the slope) using a random effects model as this will allow the effects to vary between practices. Data will be aggregated at practice level and displayed graphically. This visual inspection will inform the model by providing information on whether any change occurred at the time of the intervention or whether there was a lag in the response. We will also examine for decay or sustainability in change in prescribing after the intervention has finished. A further adjusted model will examine for potential impact of both practice and patient level factors. As it is possible that there may be partial data, the data will be examined for patterns in missingness. If it is decided that it is likely that the data are missing at random a sensitivity analysis will be carried out to model the potential impact of the missing data.

If outlying practice(s) are found, sensitivity analysis will be conducted on the primary endpoint excluding the outlying practice(s). A poorly fitting model may also lead to additional sensitivity analyses.

3.2 Secondary Analyses

Secondary objectives 1 to 4 will be analysed in the same manner as the primary analysis.

References

1. Leong, M., B. Murnion, and P. Haber, *Examination of opioid prescribing in Australia from 1992 to 2007.* Internal medicine journal, 2009. **39**(10): p. 676-681.
2. Hamunen, K., P. Paakkari, and E. Kalso, *Trends in opioid consumption in the Nordic countries 2002–2006.* European Journal of Pain, 2009. **13**(9): p. 954-962.
3. Schubert, I., P. Ihle, and R. Sabatowski, *Increase in opiate prescription in Germany between 2000 and 2010.* Deutsches Arzteblatt International, 2013. **110**(4): p. 45-51.
4. Fischer, B., W. Jones, and J. Rehm, *Trends and changes in prescription opioid analgesic dispensing in Canada 2005–2012: an update with a focus on recent interventions.* BMC health services research, 2014. **14**(1): p. 90.
5. Ruscitto, A., B. Smith, and B. Guthrie, *Changes in opioid and other analgesic use 1995–2010: Repeated cross-sectional analysis of dispensed prescribing for a large geographical population in Scotland.* European Journal of Pain, 2015. **19**(1): p. 59-66.
6. Stannard, C., *Opioids in the UK: what's the problem?* Bmj, 2013. **347**.
7. Heit, H.A. and D.L. Gourlay, *Tackling the difficult problem of prescription opioid misuse.* Annals of internal medicine, 2010. **152**(11): p. 747-748.

8. Fredheim, O.M.S., et al., *Chronic pain and use of opioids: A population-based pharmacoepidemiological study from the Norwegian Prescription Database and the Nord-Trøndelag Health Study.* PAIN®, 2014. **155**(7): p. 1213-1221.
9. Spence, D., *Bad medicine: co-codamol.* BMJ, 2013. **346**: p. f1821.
10. Ballantyne, J.C. and N.S. Shin, *Efficacy of opioids for chronic pain: a review of the evidence.* The Clinical journal of pain, 2008. **24**(6): p. 469-478.
11. Banta-Green, C.J., et al., *The prescribed opioids difficulties scale: a patient-centered assessment of problems and concerns.* The Clinical journal of pain, 2010. **26**(6): p. 489.
12. Reid, M.C., et al., *Characteristics of older adults receiving opioids in primary care: treatment duration and outcomes.* Pain Medicine, 2010. **11**(7): p. 1063-1071.
13. Solomon, D.H., et al., *The comparative safety of analgesics in older adults with arthritis.* Archives of internal medicine, 2010. **170**(22): p. 1968-1978.
14. Solomon, D.H., et al., *The comparative safety of opioids for nonmalignant pain in older adults.* Archives of internal medicine, 2010. **170**(22): p. 1979-1986.
15. Clarke, H., et al., *Rates and risk factors for prolonged opioid use after major surgery: population based cohort study.* 2014.
16. Foy, R., et al., *Prescribed opioids in primary care: cross-sectional and longitudinal analyses of influence of patient and practice characteristics.* BMJ Open, 2016. **6**(5).
17. McCrorie, C., et al., *Understanding long-term opioid prescribing for non-cancer pain in primary care: a qualitative study.* BMC Family Practice, 2015. **16**(1): p. 1-9.
18. Kalso, E., et al., *Recommendations for using opioids in chronic non-cancer pain.* European Journal of Pain, 2003. **7**(5): p. 381-386.
19. Turk, D.C., H.D. Wilson, and A. Cahana, *Treatment of chronic non-cancer pain.* The Lancet, 2011. **377**(9784): p. 2226-2235.
20. Conaghan, P.G., J. Dickson, and R.L. Grant, *Care and management of osteoarthritis in adults: summary of NICE*

guidance. British Medical Journal (BMJ), 2008. **336**(7642): p. 502-503.
21. *Opioids for persistent pain: summary of guidance on good practice from the British Pain Society.* British Journal of Pain, 2012. **6**(1): p. 9-10.
22. Dickinson, R., et al., *Long-term prescribing of antidepressants in the older population: a qualitative study.* British Journal of General Practice, 2010. **60**: p. e144-e155.
