# Supplementary material for: The effects of an evidence- and theory-informed feedback intervention on opioid prescribing for non-cancer pain in primary care: A controlled interrupted time series analysis
Source: PLoS Med. 2021 Oct 4;18(10):e1003796. doi: 10.1371/journal.pmed.1003796 (PMC8489725; doi:10.1371/journal.pmed.1003796)
Supplement: Attachment — Submitted filename: Response to reviewers.docx [file pmed.1003796.s008.docx]

*Thank you for the opportunity to revise and resubmit our manuscript. We have responded to all comments below and submitted a manuscript with changes highlighted.*

**Requests from the editors:**

*In the last sentence of the Abstract Methods and Findings section, please include a note about any key limitation(s) of the study's methodology.

*We have added the following sentence to the abstract findings section:*

*“Study limitations include being limited to one region in the UK, possible coding errors in routine data, being unable to fully account for concurrent interventions, and uncertainty whether reductions in prescribing were always clinically appropriate."*

*We have also reformatted the abstract to align with the journal style.*

*In the abstract, effects and confidence intervals are currently a bit hard to read and could be reformatted so the reader can follow these better eg:
"The number of adults prescribed any opioid rose pre-intervention in both intervention and control practices BY 0.18 (95% CI; 0.11, 0.25) and 0.36 (95% CI; 0.27, 0.46) per 1,000 adults per month respectively. During the intervention period, prescribing fell in intervention practices (change -0.11; 95% CI -0.30, -0.08) and continued rising in control practices (change 0.54; 95% CI 0.29, 0.78) with a difference of -0.65 per 1000 patients (95% CI -0.96, -0.34)"

*Thank you, we have amended the above text.*

*At this stage, we ask that you include a short, non-technical Author Summary of your research to make findings accessible to a wide audience that includes both scientists and non-scientists. The Author Summary should immediately follow the Abstract in your revised manuscript. This text is subject to editorial change and should be distinct from the scientific abstract. Please see our author guidelines for more information: <https://journals.plos.org/plosmedicine/s/revising-your-manuscript#loc-author-summary>

*We have now added an author summary to the manuscript.*

*On page 9 of the paper, currently the authors note a sensitivity analysis that isn't included with the paper (below); the journal prefers to ensure that all claims made in the paper are backed up with analyses presented and there are no page/length limits; we'd recommend that the sensitivity analysis noted below is included, with supplementary materials if needed:

"Sensitivity analysis (not shown) explored and confirmed the robustness of the modelling approaches, based on the main outcome adjusted LMM"

*We have included the sensitivity analysis in the supplementary materials (appendix 5) and referred to this in main paper.*

*On page 12, the paper references some analyses on acceptability and value of the feedback intervention (below). If the PLOS Medicine paper is accepted then it won't be possible to include a reference to a manuscript under review, and the best outcome is if the acceptability/value paper is accepted for publication by this stage (and then it can be cited and included in the reference list as "in press" - or with a full citation/DOI). Please consider this when you come to resubmit the revision, it's also possible that the way this article is cited can be updated later, if the PLOS Medicine paper gets to the stage of provisional acceptance and materials are being prepared for publication. If the acceptability/value paper isn't accepted by this point, then the authors would need to be prepared to remove any reference/citation to it.

"Our process evaluation suggests the acceptability and perceived value of credible, tailored feedback targeting issues of emerging or established concern (manuscript under review)"

*We have removed this reference to the process evaluation paper to minimise any delay in the further review and potential publication of this manuscript. Please see our response to Reviewer 2.*

*Did your study have a prospective protocol or analysis plan? Please state this (either way) early in the Methods section.

a) If a prospective analysis plan (from your funding proposal, IRB or other ethics committee submission, study protocol, or other planning document written before analyzing the data) was used in designing the study, please include the relevant prospectively written document with your revised manuscript as a Supporting Information file to be published alongside your study, and cite it in the Methods section. A legend for this file should be included at the end of your manuscript.

b) If no such document exists, please make sure that the Methods section transparently describes when analyses were planned, and when/why any data-driven changes to analyses took place.

c) In either case, changes in the analysis-- including those made in response to peer review comments-- should be identified as such in the Methods section of the paper, with rationale.

*Thank you, we have now included the statistical analysis plan within the supplementary material (Appendix 6).*

-----------------------------------------------------------

Comments from the reviewers:

**Reviewer #1:**

There is an urgent need for evidence-based interventions to reduce inappropriate opioid prescribing at both population and individual patient level and this work certainly has potential to contribute to the former category. The supplementary information regarding the intervention characteristics (TIDiER checklist) and example feedback were very helpful. The overall finding of reduced opioid prescribing in practices receiving a fairly straightforward and low cost feedback intervention is very welcome. However, 3 years have elapsed since the intervention period, during which time there have been changes in prescriber awareness and behaviour that may impact the findings and I have some reservations about how the study is reported which are summarised below.

*Thank you for your feedback. We hope that we have adequately addressed these concerns below.*

Introduction
Paragraph 1
Whilst it is true that the US has an opioid crisis - the scale of this may have peaked around 2016, based on CDC data from 2017- 18. Reference 7 is particularly old & I suggest using that to highlight that in the last 15-20 years an opioid crisis developed - in the US & other western countries before adding more up to date information from the US, UK and elsewhere that suggests the rise in prescribing may have peaked but hasn't fallen so much as may have been expected. I recommend highlighting other opioid-related harms - not just dependency e.g. overdose & falls/ fractures.

*Thank you for highlighting the increased risk of falls and fractures with opioid prescribing. This is particularly relevant to our study as we found the greatest decline in opioid prescriptions in those aged over 75 years with the intervention. We have updated the introductory paragraph to reflect this and in response to Reviewer 3’s comments.*

Paragraph 2
Line 61-62 - there is a lack of evidence for all kinds of interventions - why highlight only psychological?

*We refer to the updated Cochrane review (Eccleston C, Fisher E, Thomas KH, Hearn L, Derry S, Stannard C, Knaggs R, Moore RA. Interventions for the reduction of prescribed opioid use in chronic non‐cancer pain. Cochrane Database of Systematic Reviews 2017, Issue 11. Art. No.: CD010323.) This found little evidence for interventions in reducing opioid use, the individual studies included focused on psychological (mindfulness and CBT) interventions. We have removed the term ‘psychological’ from the statement. We have also updated this section in response to Reviewer 3 below.*

I don't think ref 16 is particularly well summarised in line 62-63 - is the struggle with managing chronic pain & the lack of effective medicines rather than with opioids per se?

*Yes, we agree with the reviewer’s assessment. However, we have reorganised this background section in response to Reviewer 3 and have taken out this point as it was not central to the argument at this point.*

Line 65 - reference 17 seems to be wrong

*Thank you for highlighting this error, we have corrected the reference.*

Methods
Study design and setting
Line 78 - whilst accepting this is worded for an international audience, it seems odd not to mention General Practice or GP practices when discussing UK primary care.

*We aimed to ensure the manuscript is understandable by an international audience, however we have added general practices here.*

I suspect controlled ITS is not a study design that many readers will be very familiar with and I think a more detailed description of that and the rationale for using it would more useful to most readers than some of the detail given about UK CCGs etc., which is to some extent repeated in the results section.

*We have removed the sentence on CCGs and added the following regarding ITS designs:*

*“CITS is a quasi-experimental design used to evaluate the longitudinal effects of interventions, through regression modelling. The addition of a control group minimises potential confounding from concurrent interventions.”*

I'm aware that there are not such clear reporting guidelines for studies using ITS design as for RCTs - but I think there are areas where more detail is needed.
How did the authors decide on the sample size - i.e. number of practices included - and what is the justification for the substantial difference in numbers of intervention and control practices?

*This study was conceived from our previous work that showed a rise in opioid prescribing in Leeds and Bradford, the two largest cities in West Yorkshire. Medicines optimisation leads for West Yorkshire CCGs asked us to deliver an intervention to reduce this prescribing, therefore our intervention sample became all practices in West Yorkshire that had a data sharing agreement with their CCG (316/317 practices). As randomisation was not possible, we conceived this CITS to determine the effectiveness of the intervention. There were difficulties in obtaining data from other CCGs within the Yorkshire and Humber area as they did not have data sharing agreements for quality improvement purposes with their practices. We worked with CCGs that agreed to share aggregated practice-level data to put data sharing agreements in place. The sample size for our control group was limited by these practicalities in obtaining data. We chose to use CCGs for the control sample in the same region as our intervention sample as these fall within the same Integrated Care System (ICS) and would be subject to the same wider initiatives and interventions regarding opioid prescribing.*

*We have acknowledged this point more briefly under Study Design and Setting by stating: ‘Our main study population, and hence sample size, was therefore limited by the coverage of the data-sharing agreements.’*

Discussion and conclusions
The study findings are reported as though they arise form an RCT rather than a quasi-experimental study design and whilst the shortcomings of the design is acknowledged to some extent, the authors conclude that "Repeated evidence and theory informed comparative feedback reversed a rising trend of opioid prescribing in primary care" but does this study design really permit such conclusions about causation?

*We have tempered this initial claim in the Discussion by adding ‘We observed that…’ However, controlled interrupted time series analysis is valuable for evaluation of health systems interventions, when RCTs are not feasible. The additional strength of using a control group helps to exclude potential confounding due to co-interventions or other events occurring around the time of the intervention. When an ITS was used to reanalyze the results of cluster RCTs, the findings were largely concordant, yielding effect estimates with overlapping 95% confidence intervals across both analytical methods: Fretheim A, Zhang F, Ross-Degnan D, Oxman AD, Cheyne H, Foy R, Goodacre S, Herrin J, Kerse N, McKinlay RJ, Wright A, Soumerai SB. A reanalysis of cluster randomized trials showed interrupted time-series studies were valuable in health system evaluation. J Clin Epidemiol. 2015 Mar;68(3):324-33. We have now cited this study under ‘limitations’ but remain confident in our study conclusions.*

Review by a methodologist and statistician is recommended - for a more expert opinion on these aspects than I can give.

-----------------------------------------------------------

**Reviewer #2:**

Thanks for the opportunity to review your manuscript. My role is as a statistical reviewer so my queries are focused on study design, data, and analysis.

This study uses a multi-level interrupted time series analysis with control sites to test the effect of an intervention intended to reduce prescription of opioid analgesics. . I have put overall queries first, and then followed by questions related to a specific section of the manuscript with a page/line reference.

*Thank you for your feedback, we hope we have adequately addressed your concerns below.*

Was there any information available in the study from the practices about whether the intervention reports were used by physicians (or others in the practices?), and what they thought of the information (i.e. acceptability)?

*We conducted a parallel process evaluation which is currently under review with a different journal. We interviewed practices and found that the feedback reports were well-received and motivating, enabling a range of responses to reduce opioid prescribing and permitting practices to respond in ways that fitted with stronger or weaker quality improvement processes and existing resources. The feedback reports did not consistently pass practice ‘gatekeeping’ processes and were sometimes lost amongst many other communications competing for attention; however, the scale, frequency and duration of feedback may have ensured a good overall level of practice population reach.*

*As we cannot yet cite the process evaluation, we now mention that how practices received and responded to the intervention will be addressed in a separate study. We therefore now highlight as a limitation of this study that it did not directly assess use and acceptability of the feedback reports.*

P6. L86. Is the same database (electronic prescribing data) used throughout the different CCGs?

*Yes, the same database and searches were used for all CCGs. We extracted (centrally) data directly from the electronic health record systems used by the practices. In Yorkshire and Humber most practices use The Phoenix Partnership SystmOne EHR and a minority use EMIS Health. Searches were designed to capture the same information from both EHR systems.*

P6. L86. How were the areas/practices that received the intervention decided? Was this random allocation or deliberate? The limitations of the study mention the quasi-experimental design but not specifically whether there were systematic differences between the intervention and control areas and whether these could lead to differing temporal changes.

*Please see our response to Reviewer 1 regarding our sample size justifications. On analysis, we found that the intervention practices were similar to controls for male to female patient ratios, patient experience scores and Quality Outcome Framework achievement (markers of quality) but had slightly larger list sizes, fewer patients with long-term conditions and had more deprived populations (Table 1). On adjustment for practice characteristics in The LMMs we found little change in the outcome estimates providing evidence that the differences between the intervention and control practices did not account for difference in outcomes.*

P6. L87. To clarify, these extra practices were in the Yorkshire + Humber region, but not in the West Yorkshire area, and not selected from the five CCGs that provide control data?

*Yes, this is correct, and we have clarified this further in the text*.

P6. L87. Is the data source for these extra practices the same as from the other sites (ie. Just a difference means for data provision)?

*Yes, all data was extracted from OpenPrescribing.com for the same dates using the same searches for this additional analysis for all intervention and control CCGs. This is detailed under “data sources and outcomes”, which we have clarified further in the text.*

P6.L87. Do patients always use the same practice? Is there any information on how many patients would move practices throughout the study period?

*Patients are registered at just one practice although may change practice if they choose. We do not have patient-level data to assess the number of patients that changed practice within the intervention year. We have been unable to find a general estimate of the number of patients that move within a year, however UK general practice populations tend to be stable year on year with limited movement between practices, mainly attributable to patients moving home in and out of practice catchment areas. It is likely that patients changing practices would potentially dilute any intervention effect rather than increase it. Unless relocating to a different area, patients would not be able to choose to move between an intervention or control practice as these are geographically situated.*

P6, L98. Were there other services available to patients in this area to replace opioid prescriptions? i.e. specialist pain management?

*Yes, but these did not form part of our intervention. We have clarified this after describing the intervention: ‘The intervention did not involve any changes to existing musculoskeletal or pain services, which general practices and patients could access as usual throughout the study period.’*

*Therefore, no increase in provision occurred in response to the intervention. We assessed referrals to musculoskeletal services to look for wider impacts on the health service as part of our analysis.*

P8. L127. Is it possible to derive total morphine equivalent dose from the prescription data? Or is lowering any opioid prescribing the main aim of the intervention, not lowering the dose or substituting a less strong agent?

*It was not possible to derive the total morphine equivalent dose from the data we had access to as we had no patient-level data. Our intervention aimed to reduce the total number of patients taking opioid medication, therefore this was our primary outcome. The reduction in strong opioid prescribing suggests the intervention was also successful in lowering the dose of opioid prescriptions. We have added to the text: ‘No patient-level data was extracted to calculate morphine equivalent doses.’*

P8. L144. This is a reasonably complicated model and I think it would be beneficial to include this in an appendix as a formula. Was a 'shift' parameter at the beginning of the intervention period used or just change over time?

*A supplementary document (Appendix 4) has now been included outlining assumptions and formula for the modelling approaches. We were not clear on what was meant by ‘shift’ parameter but assumed this referred to the interaction term that included intervention (yes/no), intervention period (pre, during, post) and month to appropriately account for the change over time in the different stages of the study as recommended by CITS guidelines (REF 34-36). We have clarified the modelling approaches in Methods to make this clearer.*

P8. L164. For which outcomes was the Poisson model used, and which for the Neg Bin? How was the decision to use these made?

*The Poisson and Neg Binomial models were compared to the LMM for the main outcome to assess that the later is the most appropriate fit to the dataset – the LMM was the best comparing AIC and BIC and so was used in all outcomes. This is now outlined in the methods and supplementary Appendix 5.*

P11, L219. Were these stratified analyses done by restricting the patients in the analysis, or by including an interaction (e.g. age x control/int variable)? It isn't detailed in the methods how this was done. Was the effect in over 75s tested directly or is this a comparison of stratified analyses?

*No stratified analysis was carried out, an interaction term was included as described above and now included in the methods and supplementary documents. The over 75s was a separate extracted outcome – now clarified in the data sources.*

Figure 1. Are these the estimated rates from the mixed models? Is it possible to also show the crude rates as well as the estimates in each month of the study? I would also consider modifying the x-axis, to make this easier to read (e.g. maybe have each year in labels and ticks for each month/quarter?)

*These are the estimates of the mixed models – now explained in the title for figure. We thank the reviewer for the useful suggestion and have amended the x-axis to make it easier to read. The crude rates would be misleading as they do not take into account the multilevel nature of the data nor the adjustment for other practice characteristics and so have not been included.*

-----------------------------------------------------------

**Reviewer #3:**

This article, "The effects of an evidence and theory-informed feedback intervention on opioid prescribing for non-cancer pain in primary care: a controlled interrupted time series analysis", has significant merit in that it is reporting a large scale, low intensity intervention and its population level impact. Because the focus was on population exposure to opioids, the primary outcome, appropriately chosen was any adult prescribed an opioid. This is essentially how many people are exposed to opioids and not necessarily any impact on people already getting opioids, particularly those with risky opioids use.

*Thank you for your feedback. We hope we have adequately addressed your comments below.*

By section:
Abstract- well written, clearly stated.

Introduction:
First paragraph-
Line 55- North America is experiencing an opioid crisis but rising opioid mortality is related more to heroin and synthetic opioids than prescription opioids. The prescription opioid crisis occurred in the 2000s-2010 after which heroin (2010) and then fentanyl (2014) took over as leading causes of opioid overdose death. Important not to promulgate misinformation about the contribution of prescription opioids to the current crisis.

*We agree and have clarified this further in the text.*

Line 58- Opioids are likely to be no more effective than non-opioid pain medication (see Krebs SPACE trial published in JAMA in 2019), which doesn't mean they are of limited effectiveness. It is important to be clear that opioids do help some people but clearly have potential for great harm and don't help everyone.

*We agree and have clarified this further in the text.*

Second paragraph;
Contrary to line 60's assertion, a number of approaches have been tested to decrease opioid prescribing. There is a literature on ECHO to reduce opioid prescribing e.g. https://www.ncbi.nlm.nih.gov/pmc/articles/PMC6420488/; also, implementation of prescription drug monitoring programs (vast literature showing decreased in population based prescribing associated with PDMP), clinical intervention to improve guideline adherent prescribing using academic detailing and nurse care managers (https://pubmed.ncbi.nlm.nih.gov/28715535/, https://pubmed.ncbi.nlm.nih.gov/32697847/) and academic detailing alone (plentiful literature on this).

*We are particularly grateful to Reviewer 3 for this point and highlighting the above studies. We now acknowledge these and have changed the contextualisation of our study in the relevant literature.*

Methods:
Study Design:
It might be useful to define "lists" in this first paragraph since this is used later in the manuscript, particularly in the tables, and it would be useful to understand how a list relates to the description of the practices for those in North America not familiar with the system in the UK.

*We have updated this section to include a description of a practice ‘list’ to make this clearer for non-UK audiences.*

Intervention:
Line 93: It would be useful to name the evidence and theory that drive the intervention. The manuscript is diminished by lack of a published protocol that could have outlined this in detail.

*The feedback reports follow the format of a previous study that developed evidence-based feedback reports that addressed the Theoretical Domains Framework determinants of adherence to quality indicators (*[*https://www.journalslibrary.nihr.ac.uk/programmes/pgfar/RP-PG-1209-10040/#/*](https://www.journalslibrary.nihr.ac.uk/programmes/pgfar/RP-PG-1209-10040/#/)*). We have added a statement to highlight this in the text.*

Data Sources and Outcomes:
The primary outcome is appropriate for this intervention. It wasn't clear why the particular high risk groups were targeted and what the goal was- For example, patients prescribed anti-depressants and opioids.

*Our earlier work highlighted that these groups of patients are at higher risk of stronger or longer courses of opioid medication (*[*https://bmjopen.bmj.com/content/6/5/e010276*](https://bmjopen.bmj.com/content/6/5/e010276)*). This work is referenced in the text detailing the intervention and we have added the reference here too for clarity.*

Was the denominator people prescribed anti-depressants and the goal to decrease opioids or was it to ensure that people prescribed opioids also be prescribed anti-depressants for concomitant depression and for analgesic impact?

*We identified in previous work that mental health diagnoses are often poorly recorded in EHRs in the UK, reflecting how general practitioners pragmatically prescribe according to symptoms and responses to treatment rather than diagnostic categories (*[*https://bmjopen.bmj.com/content/4/8/e005178*](https://bmjopen.bmj.com/content/4/8/e005178)*). We used co-prescription of an anti-depressant as a further measure of patients with mental health problems and at higher risk of stronger and longer opioid courses.*

*The denominator for all outcomes was the number of adults per practice per month, except for the rate of adults aged >75 years prescribed opioid, where the number of adults over 75 per practice per month was used – this is now clarified in the Data Sources and Outcomes section.*

Similarly, the strong vs. weak opioid analysis wasn't clearly justified although it more intuitive. Was it based on Morphine Equivalent doses? If so, the reporting on the data might include that, since someone one a weak opioid might be prescribed high amounts and someone on a stronger opioid might be prescribed just a few tablets. In the US, hydrocodone is one of the most commonly prescribed opioids but this was not listed here. Perhaps that is due to a different name in the UK or that it isn't on the formulary.

*We categorised opioids according to World Health Organization reported potency (https://apps.who.int/iris/bitstream/handle/10665/37896/9241544821.pdf;jsessionid=90928327C3AF967A48C30C17E412015F?sequence=1) and have updated the text to make this clearer. We agree that the Morphine Equivalent dose for some patients may be higher with taking multiple lower potency opioids than someone taking fewer stronger opioids, however as detailed above, we were unable to access patient-level data to calculate this.*

*Hydrocodone is not licensed within the UK so is not prescribed or included in our analysis.*

Lastly, excluding individuals with drug dependence may exclude people prescribed opioids and then later diagnosed with drug dependence as a result of the prescription.

*We agree, however within UK primary care some practices are specialist substance abuse treatment centres as well as providing general medical services. As we did not want to penalise practices which undertake this work, we decided to exclude patients with drug dependency from our feedback reports and therefore the analysis. This limitation is acknowledged in our discussion.*

The use of gabapentin and NSAID was useful to include as a comparator.
*Thank you.*

Data Analysis-
A statistician should comment on these analyses, but to a non-statistician, this looks complete and well detailed.
The discussion reports on cost estimates, but there is no description where those calculations come from.

*The estimate is based on known costs (e.g. postage, data extraction fees) and time spent by staff (full time equivalent salaries). This is an estimate rather than a full cost-analysis and so we have reported this as such. We have updated the Methods, Results and Discussion sections to reflect this.*

See comment below on results/tables

Results:
The reporting of the results in the body of the manuscript seem clear but don't seem to match up with the values in the tables, which were a bit confusing. For example, the results in table 3 showed two values- the beginning of the period and the end of the period. But in some cases, the numbers didn't make sense. For example, number of adults prescribed opioids per 1000 control group 2016m3 was 68.2 but the next month 2016m4 was 63.9. Why was there such a disparity month to month? The same with the figure- it looks like it drops precipitously in a month period. Why wasn't this displayed for a rolling average? It might not be right to display this way, but in Larochelle, 2016 Annals of Internal Medicine, appendix figure 2, we showed a daily average before and after an index event, which was steadier over time, reflecting the reality of opioid prescribing. This article might have something similar but using an 8 week rolling average of adults per 1000 given opioids.
The fact that the data is displayed this way makes me question whether all months of data were used, and if so, why weren't they incorporated into tables and figures?

*All months data were included in the models and the estimates for each are shown in Figure 1 (the jumps in the figure have been removed to avoid confusion). Each months’ estimates were not shown in the tables and it is recommended for CITS to show for each of the three phases - the starting and ending values and the change over each phase. The nature of CITS means that we estimate the 3 phases as three ‘regression’ line which can result in these jumps. The multi-level model is the most appropriate approach to take into account the correlation between outcomes over time for each practice and the variation between practices which can be a limitation of a moving average.*

It wasn't clear why the authors did an uncontrolled Interrupted time series of the intervention practices alone and it doesn't augment any of the findings or discussion in the manuscript. This is superfluous data analysis.

*This is a recommendation of CITS analysis to include the analysis of the intervention practices only as a simple ITS. The results of the simple ITS (Table 5) mirrors that of the CITS (Table 3), providing greater confidence that any association between intervention and the effect is likely to be causal, and evidence that the control practices did not experience some other event. This is now expanded on in the results section.*

Cost estimates should go in Results section along with details of how they were calculated prior to the discussion.

*We have done this as mentioned above.*

Discussion:
Paragraph 1- it would be useful to show the estimates of the population impact earlier, in the results

*We have moved this sentence to the results and discussed the potential for population impact in the discussion instead.*

Paragraph 2- line 216-17 Since there was no analysis of MME, just the type of opioid, it isn't at all clear why there would need to be a gradual reduction to limit withdrawal symptoms. Most people on moderate doses would not experience withdrawal.

*Previous studies have shown the majority of patients taking strong opioids within the UK tend to have had dose escalation from weaker and lower MME and are likely to have been taking a high MME for a longer period (*[*https://doi.org/10.1371/journal.pmed.1003270*](https://doi.org/10.1371/journal.pmed.1003270)*), they are therefore likely to need a longer de-prescribing process than those taking weaker and lower MME doses. This study showed that in the UK, it is rare to start a patient on a strong opioid without trying a weaker opioids first and increasing the dose.*

*We have qualified our statement here given that it involved making an assumption about the nature of de-prescribing: ‘The number of patients prescribed strong opioids fell during the intervention, although at a slower rate possibly reflecting a longer de-prescribing process than for weaker opioids, given the need for gradual reductions to limit withdrawal symptoms.’*

Paragraph 3- the reduction of non-opioid medication and referrals may have had positive financial impact, but it may be that practitioners are leaving patients without any treatment for pain. There was no evidence of self-management options in the data reported. Furthermore, it generally takes longer to work with patients on self-management than a prescription medication so it is hard to know what actually occurred. Best to leave out speculation and do some future studies examining what did happen for patients with pain.

*We conducted a parallel process evaluation to understand what happened in practice and this suggested that prescribers discussed self-management options with patients. As mentioned in our response to Reviewer 2, we now highlight as a limitation of this study that it did not directly assess use and acceptability of the feedback reports. We acknowledge that this point remains speculative in this manuscript but suggest that it may help readers in considering possible downstream impacts of the feedback intervention.*

Paragraph 5- see comment above about presenting cost data in the discussion without presenting first in the results. That being said, this has important implications for the intervention's impact on the population and health service.

*We have answered this comment above.*

Paragraph 10 (lines 273-276) The lack of information on what actually happened in the clinical interaction with the patient is one of the most important limitations of the study. The generalizability and coding errors were expected as part of the study but would be balanced by the large size and reach (generalizability) as well as balance between intervention and control (coding errors). What we don't know is how the patients fared with the intervention. The lack of referrals and non-opioid medications is not necessarily reassuring because it could indicate that the pain was not addressed at all.

*As mentioned above, we have undertaken a process evaluation to look at how practices responded to the feedback intervention which we hope to publish shortly. We now acknowledge this as a limitation to this study.*

Tables:
Table 1:
It would be important to define some of the headings in expanded footnotes below, including:
List
Patient Experience- what does median patient experience mean- what measure is used and what is the scale?
LTC- what are included in these?
QoF- What does % score mean?
IMD- What does % IMD indicate?

*Thank you, we have now defined these further in the footnotes.*

Table 2:
Baseline characteristics- are these a 12 month average? One month average? The time frame needs to be defined.
CCG should be defined in footnote of table

*The baseline characteristics are the first month average of the data collection. We have updated this in the table and added the CCG definition.*

Table 3:
See critique above-
Also, this table is very busy with lots of detail. It would be useful to simplify, if possible. Perhaps show mean numbers for the pre-intervention, intervention and post-intervention periods rather than the multiple data points.

*As we mentioned above in response to the comment on the results, it is recommended for CITS to show for each of the three phases - the starting and ending values and the change over each phase in both the control and intervention groups – just showing the change and difference in change for each phase is only part of the trajectory. Whilst this means the table has more detail, it conforms to current best practice in ITS reporting.*

Table 4:
Similar critique as for Table 3- could simplify it

*This is answered above for table 3.*

In summary, this is an important intervention study with powerful population level results. It could use improvement in each of the main section of the paper and in the display of data.
