# Supplementary material for: The effects of an evidence- and theory-informed feedback intervention on opioid prescribing for non-cancer pain in primary care: A controlled interrupted time series analysis
Source: PLoS Med. 2021 Oct 4;18(10):e1003796. doi: 10.1371/journal.pmed.1003796 (PMC8489725; doi:10.1371/journal.pmed.1003796)
Supplement: Attachment — Submitted filename: Response to Reviewers 2.docx [file pmed.1003796.s009.docx]

**Response to reviewers:**

Thank you for the further opportunity to improve the clarity of our manuscript.

**Requests from Editors:**

**Comments from Reviewers:**

**Reviewer #1: The authors have taken on board reviewer feedback. As a result, the revised manuscript is much improved and reads well - thank you! I have no further suggestions**
Thank you for your review.

**Reviewer #2: Thanks for the opportunity to see your revised manuscript. Overall I think that the changes in the version resolve all of my queries from the first version I reviewed. The data sources section is clearer and the inclusion of the SAP was helpful for me as well.**
**The supplementary material was a useful addition. I can now follow the analysis with the equation for the main LMM model displayed. The Model fit table (S2) supports the use of the LMM and the results of the sensitivity analysis are similar during the pre and intervention periods. The post-intervention change over time seems to shift in the intervention areas, becoming positive whereas before it declined (although it's still lower than the control areas). The numbering of these tables may need to be adjusted as Table S1 follows Table S2 in the appendix.**
**I think the information you provided about how the study groups were formed (request from staff at practices that eventually received the intervention, and then surrounding area) should be presented in the methods.**
**This is a nice study - clearly lots work went into this and it has resulted in a good manuscript.**
Thank you for your review. We have updated the table numbers in the supplementary information.


**Reviewer #3: Overall, this is a much improved manuscript. It reads more clearly and was very responsive to the reviewer comments. I was not familiar with CITS and it was helpful to get the explanations provided in the response to reviewers, particularly about interpretation of the tables.**

Thank you for your further review. We hope we have clarified the points below in the text.

**There are a few things remaining that would be useful to clarify.**

**It was not clear to me until reading the response to the reviewers that this study took advantage of a natural experiment to examine the impact of a public health/health policy-driven intervention to address a rise in opioid prescribing in Leeds and Bradford by conducting a quasi-experimental analysis. This might be of interest to the readers, which also puts into context the the entire project. While statisticians are likely to understand that a controlled interrupted time series is quasi-experimental, many readers who are interested in clinical interventions for opioids prescribing may not understand this. Thus, I suggest that it be clarified in the methods section that the intervention was implemented in targeted areas and the control sites were identified to match the region and other characteristics of the intervention site. And it might be useful to add the term quasi-experimental in the abstract to alert readers unfamiliar with controlled interrupted time series.**
**In my mind, the quasi experimental design is an important limitation as these sites were aware on some level that they were receiving this intervention because of poor performance. While the analysis makes a convincing argument that the intervention was impactful, there may be other forces at play. This should be added to the limitations.**
We have updated the study design and setting section to include this information:

“Yorkshire and Humber region covers an ethnically diverse population of 5.4 million residents with above average socioeconomic deprivation levels(23, 24). This study was conceived from our previous work that showed a rise in opioid prescribing in Leeds and Bradford, the two largest cities in West Yorkshire. Medicines optimisation leads for West Yorkshire asked us to deliver an intervention to reduce this prescribing in this area. West Yorkshire (intervention group) has a population of 2.2 million residents served by 317 practices organised within 10 clinical commissioning groups (CCGs) in 2016. One practice declined data sharing for this study. Five CCGs from the wider Yorkshire and Humber region (outside of West Yorkshire), with a population of one million residents and 187 practices, provided control data. We chose to use CCGs for the control sample in the same region as our intervention sample as these would be subject to similar region-wide prescribing initiatives. Our main study population, and hence sample size, was therefore limited by the coverage of the data-sharing agreements.”

We have added the term ‘quasi-experimental’ to the abstract methods.

The limitations of the quasi-experimental design are discussed in the limitations and we have added that the publication of our previous study documenting the rise in prescribing took place concurrently (intervention started April 2016, publication May 2016):

“Third, the quasi-experimental design cannot fully account for concurrent interventions. The concurrent publication showing the rise in prescribing in this area may have alerted practices to rising opioid prescribing.”

**There is some confusion about the number of control practices. In the abstract, it says 130 control practices. In the Author Summary it says 187 practices. In the methods, on line 130, it says 187 practices provided control data, and on line 132 it was 134 practices were added as controls**.
Thank you for this, we had not spotted the inconsistency. We have changed the author summary to 130 practices as this was the number used in our primary outcome. The addition of a further 134 practices on line 132 were for the analysis of publicly available prescribing data.

**In my prior review, I may not have been clear about my question about anti-depressants. If I understand the response to reviewers, anti-depressants were used as a proxy for mental illness in addition to analysis of diagnoses of mental illness. It might be helpful to be explicit about that in the manuscript. Other studies of pain control have used anti-depressants as a specific intervention for chronic pain, so higher antidepressant may be interpreted as a therapeutic intervention, not a proxy for mental illness**.

We have added this clarification to the text.

**Tables: table 1 much improved- it would be useful to put the explanations below the table in order that the columns appear in the table. Patient experience is currently at the bottom despite being the 5th column.**

We have adjusted the order of the explanations to reflect the table columns.

**Minor typo- line 278 used the Pound rather than Dollar sign.**
We have amended.
